# The Relationship Between Social Determinants of Health and Cholesteatoma Care

**DOI:** 10.1097/ONO.0000000000000069

**Published:** 2025-04-17

**Authors:** Mayuri S. Patel, Estephania Candelo, Alexander Hochwald, Mallory Raymond

**Affiliations:** 1 Department of Otolaryngology-Head and Neck Surgery, Mayo Clinic Florida, Jacksonville, FL; 2 Department of Quantitative Health Sciences, Mayo Clinic, Florida, Jacksonville, FL

**Keywords:** Cholesteatoma, Disparities, Otology, social determinants of health

## Abstract

**Objective:**

To explore the associations between social determinants of health (SDOH) and the access to, delivery of, and outcomes of cholesteatoma care.

**Study Design:**

Retrospective review.

**Setting:**

Multisite tertiary care institution.

**Methods:**

Seventy-five adults (aged 18–83) with cholesteatoma who completed SDOH questionnaires were included. Outcome measures included SDOH risk factor prevalence (stress, housing instability, financial resource strain, social connectedness, food insecurity, intimate partner violence), disease severity, hearing loss degree at presentation, mean time from symptom onset to diagnosis, postoperative improvements in air-bone gaps (ABG), complication rates, and two-stage surgery rates.

**Results:**

Stress (n = 45; 60%) was the most prevalent SDOH risk factor. Females (n = 23; 79.3%; *P* = 0.05; d = –0.24]), younger adults (mean age = 45; *P* = 0.01; d = –0.69), and individuals with lower education (n = 23; 82.1%; *P* = 0.04; d= –0.26) were more likely to have at least one SDOH risk factor. Patients with SDOH risk factors had better preoperative air pure tone averages (mean [SD] = 39.7 [16]; *P* = 0.01; d = –0.69) than patients without SDOH risk factors (mean [SD] = 50.1 [13.5]); but the ABG was similar between groups (*P* = 0.16; d = –0.38). No differences were identified in any measure of cholesteatoma care between patients with and without SDOH risk factors.

**Conclusion:**

Stress is a prevalent SDOH risk factor among patients with cholesteatoma, but it might not influence cholesteatoma care. Though female sex, younger age, and lower education levels were associated with having at least one SDOH risk factor, these also might not influence cholesteatoma care. Intentional study of larger, more heterogeneous populations is necessary to validate these observations.

Cholesteatoma is an otologic condition characterized by the accumulation of exfoliated keratin and squamous epithelium debris in the middle ear or mastoid space, with a prevalence of 9–12.6 cases per 100,000 adults (1). This abnormal growth of keratin debris is locally invasive and can compromise the integrity of the middle ear and mastoid structures, leading to otorrhea (66.5%), otalgia, and hearing impairment (7.6%) (2–4). Audiologic evaluation is utilized to identify the degree and type of hearing loss. Tympanomastoidectomy is recommended to achieve a safe, dry ear and to potentially preserve or restore hearing (5). Untreated cholesteatoma can lead to hearing loss, facial paralysis, labyrinthine fistula, and intracranial complications (4). Consequently, providing timely and appropriate care to patients is crucial.

Social determinants of health (SDOH) encompass a broad spectrum of demographic and social factors that exert a significant influence on the emergence of diseases, healthcare accessibility, patient adherence to treatment regimens, and overall health outcomes (6). Although there is an increasing interest in examining the relationship between SDOH and healthcare access, delivery, and outcomes, research specifically targeting the association between SDOH and cholesteatoma care is limited. Recent systematic reviews have indicated that certain social factors, such as zip code or low socioeconomic status, might affect the number of surgeries, rates of postoperative residual cholesteatoma, and surgery invasiveness (7,8). Age, gender, and ethnicity have also been variably associated with treatment results, but the extent to which other SDOH factors influence cholesteatoma care remains unclear (9). Additionally, recent studies have investigated the relationship between area deprivation indices and delays in care or disease severity of various otologic conditions (10,11), but these indices may not capture the complexities of SDOH in some communities (12).

To date, the specific domains of stress, housing instability, financial resource strain, social connectedness, food insecurity, and intimate partner violence that are often included in area deprivation indices and their influence on cholesteatoma care have not been studied. However, in other areas of healthcare, many of these specific domains have been shown to influence mortality, healthcare utilization, and medication adherence (13,14). Given the chronic, somewhat indolent nature of cholesteatoma, it could be theorized that patients lacking housing stability, financial stability, social connectedness, or accessible transportation may experience delays in care, present with more advanced disease, or be at higher risks of complications. Additionally, these SDOHs have the potential to bias providers in management plans. Therefore, in this study, we aimed to investigate the association between stress, housing instability, financial resource strain, social connectedness, food insecurity, and intimate partner violence on the access to, delivery of, and outcomes in cholesteatoma care, with the goal of providing data that can help address possible inequities in cholesteatoma care.

## MATERIALS AND METHODS

### Data Source

The Mayo Clinic Institutional Review Board (IRB: 22-01310) approved this multisite retrospective review study examining the relationship between SDOH and access to, delivery of, and outcomes in cholesteatoma care. The study included all patients seen at any Mayo Clinic location (Florida, Arizona, and Minnesota) between 2021 and 2023. Data collection began in 2021 and continued in the referenced format until May 2023. All data were collected through the EPIC Electronic Medical Record system and recorded in the Research Electronic Data Capture (REDCap) platform.

### Study Population

Our study population was restricted to adult patients (≥18 years old) diagnosed with cholesteatoma who underwent either primary or revision surgery. A total of 237 adult patients met the inclusion criteria. Among these, 5 patients were identified with bilateral cholesteatoma, and only data from their most recently affected ear were included in the analysis. Patients were excluded if they were under 18 years of age, acquired cholesteatoma due to physical trauma to the ear, did not undergo surgical intervention within the study timeframe, or did not complete a preoperative SDOH questionnaire (Supplemental Table 1, http://links.lww.com/ONO/A36).

### Data Collection and Outcome Definition

Data collection included patient demographics, SDOH risk factors at the time of diagnosis and the last visit, pre- and postoperative audiometry, disease severity, surgical approach, and complication rates. Patient demographics included sex, race, ethnicity, marital status, insurance status, education, language, tobacco use, alcohol use, depression history, and diagnosis age. Additionally, patient residence was categorized using the 2013 Rural-Urban Continuum Codes, distinguishing between urban and rural areas (15).

SDOH questionnaires (Supplemental Table 1, http://links.lww.com/ONO/A36) were integrated into Mayo Clinic’s standard intake process for all patients in June 2019 and addressed the topics of social connections, tobacco use, depression, physical activity, transportation needs, housing stability, alcohol use, financial strain, stress, food insecurity, and intimate partner violence. Questionnaires were distributed to patients every 6 months. The questionnaire responses closest to the time of cholesteatoma evaluation and before surgery were utilized. Responses were recorded in a raw format and then converted into categorical degrees of risk—low, medium, or high—as detailed in Supplemental Table 1, http://links.lww.com/ONO/A36. Subsequently, these categories were translated into 2 binary variables for analysis: “risk factor present” for patients with moderate or high-risk levels and “risk factor not present” for those with no risk or low degree of risk.

Proxy data points collected to assess care included the duration from symptom onset to diagnosis, alongside 3 measures of disease severity: (1) whether cholesteatoma involved the mastoid cavity, (2) the presence of ossicular chain erosion or discontinuity at the time of surgery, and (3) the identification of disease persistence during the follow-up period. For evaluating the delivery of care, a single proxy data point distinguished between single versus two-stage surgeries. The outcomes of cholesteatoma care were measured by the pre- to postoperative changes in air and bone pure tone averages (PTAs), the closure of air-bone gaps (ABG), and complication rates. Air and bone PTAs were calculated from thresholds at 500, 1000, 2000, and 4000 Hz pure tones, and ABGs were derived from the difference between them.

### Statistical Analysis

Despite the diagnosis of cholesteatoma in 237 patients, only 75 patients had a complete set of SDOH data at the time of diagnosis. Descriptive and comparative analyses were conducted using R software. The Shapiro-Wilk test, utilized to assess normality, indicated a non-normal distribution. As a result, continuous variables were summarized using median and interquartile range (IQR) where appropriate. For the analysis of non-normally distributed data, nonparametric tests, including the Mann–Whitney *U* test and the Kruskal–Wallis test, were utilized. Confidence intervals were set at 95%, and all *P* values were reported as two-sided, with a significance threshold established at a *P* value of less than 0.05. Effect sizes were determined using Cohen’s d, with the size of the effect categorized as small (0.2–0.49), medium (0.5–0.79), and large (0.8 or greater) (16).

## RESULTS

Among the 75 patients included in the study, the median age was 51 years (range = 18–83) (Table 1). Of these, 46 (61.3%) were males, and 71 (94.6%) identified as white. More than half of the patients were married (n = 44; 58.6%) and had private insurance (n = 46; 61.3%). Approximately 66.7% (n = 50) of the patients resided in urban areas.

**TABLE 1.** Demographic, educational, and social characteristics stratified by social determinants of health risk factors

	No SDOH (n = 26)	SDOH (n = 49)	Total (n = 75)	*P*	Cohen’s d or risk difference
Age of diagnosis^a^	61 (30–83)	45 (18–76)	51 (18–83)	0.01	–0.69 (–1.18 to –0.19)
Sex					
Female	6 (20.7%)	23 (79.3%)	29 (38.6%)	0.05	–0.24 (–0.26 to –0.22)
Male	20 (43.5%)	26 (56.5%)	46 (61.3%)		
Race					
White	24 (33.8%)	47 (66.2%)	71 (94.7%)	0.61	–0.0.4 (–0.08 to 0.01)
Nonwhite	2 (50%)	2 (50%)	4 (5.3%)		
Marital status					
Married	17 (38.6%)	27 (61.4%)	44 (58.6%)	0.46	0.10 (0.06–0.15)
Not married	9 (29%)	22 (71%)	31 (41.3%)		
Education					
High school or less	5 (17.9%)	23 (82.1%)	28 (37.3%)	0.04	–0.26 (–0.29 to –0.23)
More than high school	18 (41.9%)	25 (58.1%)	43 (57.3%)		
Unknown	3 (11.5%)	1 (2.1%)	4 (5.3%)		
Insurance					
Private	16 (34.8%)	30 (65.2%)	46 (61.3%)	1.00	0.02 (–0.04 to 0.07)
Government	9 (33.3%)	18 (66.7%)	27 (36%)		
Unknown	1 (3.8%)	1 (2.1%)	2 (2.6%)		
Tobacco use					
Current tobacco use	5 (41.7%)	7 (58.3%)	12 (16%)	0.74	0.14 (0.10–0.19)
No current tobacco use	20 (33.9%)	39 (66.1%)	59 (78.6%)		
Unknown	1 (3.8%)	3 (6.1%)	4 (5.3%)		
Alcohol use					
Current alcohol use	16 (34%)	31 (66%)	47 (62.6%)	0.99	–0.02 (–0.07 to 0.03)
No current alcohol use	10 (35.7%)	18 (64.3%)	28 (37.3%)		
Depression history					
Yes	2 (13.3%)	13 (86.7%)	15 (20%)	0.07	–0.21 (–0.13 to –0.23)
No	24 (42.1%)	33 (57.9%)	57 (76%)		
Unknown	0	3 (6.1%)	3 (4%)		
Area					
Urban	18 (36%)	32 (64%)	50 (66.6%)	0.61	0.07 (0.02–0.11)
Rural	7 (29.2%)	17 (70.8%)	24 (32%)		
Unknown	1 (3.8%)	0	1 (1.3%)		
Time from symptom onset to diagnosis (months)^a^	2.5 (0–75)	4 (0–84)	4 (0–84)	0.53	0.12 (–0.36 to 0.61)

a Median and Range.

SDOH, social determinants of health.

Forty-nine patients reported having at least one SDOH risk factor. Stress was the most prevalent SDOH risk factor identified at the time of diagnosis, identified in 45 (60%) patients, followed by housing instability for 6 (8%), financial resource strain for 4 (5.3%), poor social connections for 4 (5.3%), food insecurity for 3 (4%), and intimate partner violence for 1 (1.3%) (Table 2). Statistical analysis revealed no significant differences between living areas (urban versus rural) and the presence of one or more SDOH risk factors (Table 1). However, females (n = 23; 79.3%) were more likely than males (n = 26; 56.5%; *P* = 0.05; d = –0.24) to have one or more SDOH risk factors (Table 1). Patients with one or more SDOH risk factors were, on average, younger (mean age = 45; range [18–76 years] versus mean age 61; range [30–83 years]; *P* = 0.01; d = –0.69) and more likely to have achieved an educational level of high school or less (n = 23; 82.1% versus n = 5; 17.9%; *P* = 0.04; d= –0.26) compared to patients without SDOH risk factors (Table 1).

**TABLE 2.** Social determinants of health profile at diagnosis

SDOH category	Distribution of degree of SDOH risk	Distribution of binary SDOH risk (n = 75)
Social connections	NA	Yes (n = 71; 94.7%) No (n = 4; 5.3%) Unknown (n = 0; 0%)
Transportation needs	Low (n = 0; 0%) Medium (n = 0; 0%) High (n = 0; 0%)	Yes (0; 0%) No (75; 100%) Unknown (0; 0%)
Housing stability	Low (n = 0; 0%) Medium (n = 0; 0%) High (n = 7; 100%)	Yes (69; 92%) No (6; 8%) Unknown (0; 0%)
Financial resource strain	Low (n = 0; 0%) Medium (n = 2; 50%) High (n = 2; 50%)	Yes (4; 5.3%) No (71; 94.7%) Unknown (0; 0%)
Food insecurity	Low (n = 0; 0%) Medium (n = 0; 0%) High (n = 3; 100%)	Yes (3; 4%) No (72; 96%) Unknown (0; 0%)
Intimate partner violence	Low (n = 0; 0%) Medium (n = 0; 0%) High (n = 1; 100%)	Yes (1; 1.3%) No (74; 98.7%) Unknown (0; 0%)
Stress	Low (n = 26; 57.8%) Medium (n = 13; 28.9%) High (n = 6; 13.3%)	Yes (45; 60%) No (30; 40%) Unknown (0; 0%)

SDOH, social determinants of health.

Patients with at least one SDOH risk factor had better hearing (air PTA mean [SD] = 39.7 [16]) at the time of diagnosis than those without SDOH risk factors (air PTA mean [SD] = 50.1 [13.5]; *P* = 0.01; d = –0.69) (Table 3). However, no significant differences were noted in ABG between patients with or without SDOH risk factors (mean [SD] = 22.11 [9.2] versus 25.6 [8.9]; *P* = 0.16; d = –0.38) (Table 3). Similarly, postoperative ABGs did not significantly differ between patients with and without SDOH risk factors (mean [SD] = 21.1 [11] versus 23.3 [12]; *P* = 0.54; Cohen’s d = –0.19) (Table 4).

**TABLE 3.** Comparative audiometric results at diagnosis: patients with and without social determinants of health risk factors

	No SDOH at diagnosis (n = 26)	SDOH at diagnosis (n = 49)	Total (n = 75)	*P*	Cohen’s d or risk difference
Air PTA, mean (SD)	50.13 (± 13.48)	39.7 (± 16.04)	43.31 (± 15.91)	0.01	–0.69 (–1.21 to –0.16)
Air-bone gap, mean (SD)	25.6 (± 8.86)	22.11(± 9.2)	23.29 (± 9.16)	0.16	–0.38 (–0.92 to 0.16)
Bone PTA, mean (SD)	27.25 (± 15.23)	18.94 (± 13.97)	21.91 (± 14.87)	0.03	–0.58 (–1.1 to –0.06)

PTA, pure tone averages; SDOH, social determinants of health.

**TABLE 4.** Postoperative audiometric outcomes comparing patients with and without social determinants of health risk factors

	No SDOH at diagnosis (n = 26)	SDOH at diagnosis (n = 49)	Total (n = 75)	*P*	Cohen’s d or risk difference
Air PTA, mean (SD)	47 (± 18.39)	38.47 (± 16.43)	41.82 (± 17.58)	0.089	–0.50 (–1.09 to 0.09)
Air-bone gap, Mean (SD)	23.28 (± 12)	21.13 (± 10.99)	21.94 (± 11.23)	0.54	–0.19 (–0.81 to 0.43)
Bone PTA, Mean (SD)	26.09 (± 12.53)	20.03 (± 13.67)	22.28 (± 13.47)	0.11	–0.46 (–1.03 to 0.12)

PTA, pure tone averages; SDOH, social determinants of health.

The time from symptom onset to diagnosis was comparable between patients with and without SDOH risk factors (median [IQR] = 2.5 [0–75] versus 4 [0–84] months; *P* = 0.53) (Table 1). Similarly, there was no difference in total mean follow-up (from diagnosis to last follow-up) between patients with and without SDOH (mean (SD) = 3.6 (2.7) versus 3.8 (2.5); *P* = 0.78) (Table 6). Complications, including otorrhea, otalgia, tinnitus, dizziness, imbalance, aural fullness, and dysgeusia, were generally mild with no significant differences in complication rates observed between the 2 groups (n = 16 [33.3%] versus n = 4 [15.4%]; *P* = 0.11) (Table 6). Furthermore, no significant differences were identified in rates of infections (n = 1 [2.1%] versus n = 1 [3.8%]; *P* = 1.00), mastoid involvement (n = 17 [34.7%] versus n = 11 [42.3%]; *P* = 0.62), intact ossicular chains (n = 20 [40.8%] versus n = 10 [38.5%]; *P* = 0.56), and cholesteatoma persistence in the follow-up period (n = 2 [4.4%] versus n = 1 [4.2%]; *P* = 1.00) between patients with and without SDOH risk factors (Table 5 and 6). Additionally, rates of undergoing 2-staged surgeries were similar between the groups (n = 5 [10.2%] versus n = 4 (15.4%); *P* = 0.71) (Table 6).

**TABLE 5.** Disease severity analysis by social determinants of health risk factors

	Cholesteatoma involving mastoid			
	No SDOH (n = 26)	SDOH (n = 49)	Total (n = 75)	*P*
Yes	11 (42.3%)	17 (34.7%)	28 (37.3%)	0.62
No	15 (57.7%)	32 (65.3%)	47 (62.7%)	
Unknown	0 (0%)	0 (0%)	0 (0%)	
	Ossicular chain intact			
	No SDOH (n = 26)	SDOH (n = 49)	Total (n = 75)	*P*
Yes	10 (38.5%)	20 (40.8%)	30 (40%)	0.56
No	15 (57.7%)	29 (59.2%)	44 (58.7%)	
Unknown	1 (3.8%)	0 (0%)	1 (1.3%)	
	Recurrence or persistence in follow-up window			
	No SDOH (n = 24)	SDOH (n = 45)	Total (n = 69)	*P*
Yes	1 (4.2%)	2 (4.4%)	3 (4.3%)	1.00
No	23 (95.8%)	43 (95.6%)	66 (95.7%)	
Unknown				

SDOH, social determinants of health.

**TABLE 6.** Outcomes of surgery stratified by social determinants of health risk factors

	Second stage			
	No SDOH (n = 26)	SDOH (n = 49)	Total (n = 75)	*P*
Yes	4 (15.4%)	5 (10.2%)	9 (12%)	0.71
No	22 (84.6%)	44 (89.8%)	66 (88%)	
	Complications from surgery			
	No SDOH (n = 26)	SDOH (n = 48)	Total (n = 74)	*P*
Yes	4 (15.4%)	16 (33.3%)	20 (27%)	0.11
No	22 (84.6%)	32 (66.7%)	54 (73%)	
	Infections			
	No SDOH (n = 26)	SDOH (n = 48)	Total (n = 74)	*P*
Yes	1 (3.8%)	1 (2.1%)	2 (2.7%)	1.00
No	25 (96.2%)	47(97.9%)	72 (97.3%)	
	Length of follow-up, months (time from diagnosis to last visit)^a^			
	No SDOH (n = 26)	SDOH (n = 49)	Total (n = 75)	*P*
	3.6 (2.7)	3.8 (2.5)	3.7 (2.5)	0.78

a Mean (SD).

SDOH, social determinants of health.

## DISCUSSION

SDOH factors have been studied in various otolaryngological conditions, including chronic rhinosinusitis, olfactory dysfunction, head and neck cancers, vestibular schwannoma, and more recently, hearing loss and post-tympanostomy tube otorrhea (17–21). These studies consistently demonstrate that patients of lower socioeconomic status, belonging to minority ethnic backgrounds, with lower educational levels, engaging in unhealthy lifestyle factors, and those without private insurance, might face increased odds of care delays due to transportation barriers and receive suboptimal and lower-quality care compared to patients without similar demographic profiles. This study is the first to explore the impact of previously unstudied SDOH risk factors on the access to, delivery of, and outcomes of cholesteatoma care. In our cohort, we found no association between the presence of at least one SDOH risk factor and any of the primary outcome measures of the study. This finding diverges from other research that had previously suggested that rates of postoperative residual cholesteatoma, disease severity at presentation, and the invasiveness and number of surgeries performed may be influenced by social factors (7,8).

In this cohort, however, we identified several demographic factors associated with having at least one SDOH risk factor: female sex, education level, and age. While the associations between female sex and SDOH risk factors in patients with cholesteatoma remain uncertain, existing research highlights noteworthy gender-based disparities. Specifically, females have been found to exhibit a higher propensity for low-income status, screen positive for depression, underutilize healthcare, and be unpartnered compared to male counterparts (22,23). Our study also contributes valuable evidence supporting the notion that younger adults are disproportionately affected by SDOH risk factors. This observation is in line with prior literature, indicating a higher prevalence of chronic stress and financial concerns among younger adults compared to older adults (24,25). Education level is considered an important SDOH because it affects income, which in turn influences other SDOH factors. Higher educational levels are associated with lower unemployment rates and are strongly linked to better health outcomes and reduced mortality (26). Adults with less education often report poorer general health and a higher number of chronic illnesses (27). Research has indicated a greater degree of hearing loss and a higher prevalence of chronic otitis media in patients with low health literacy (28,29). Identifying whether individuals with lower educational attainment are at an increased risk of developing cholesteatoma represents a critical area for further research.

Given that 60% (n = 45) of patients in this cohort self-reported stress as an SDOH risk factor, our findings comparing groups with and without SDOH risk factors primarily reflect the influence of stress. Our analysis revealed no statistical differences in disease severity or outcomes between patients with and without SDOH risk factors, suggesting that stress likely does not substantially impact the access to, delivery of, and outcomes in cholesteatoma care. However, existing research on stress’s impact on other otologic conditions, such as tinnitus and idiopathic sudden sensorineural hearing loss, indicates a potential influence on symptom severity and onset, albeit likely through biological mechanisms (30,31). While stress is not expected to biologically affect cholesteatoma disease severity, it could hypothetically lead to delays in seeking care or nonadherence to postoperative care protocols, potentially affecting disease severity and outcomes. It is, therefore, reassuring that our study did not identify such a relationship.

The lack of association found between rurality of residence, disease persistence in the follow-up window, and complication rates and SDOH risk factors differs from several existing studies. For instance, a study on SDOH and vestibular schwannoma reported a higher tumor grade at presentation in areas with a higher deprivation index (10). Additionally, a significant delay was observed from the onset of hearing loss to cochlear implantation among patients in rural areas (32). Our findings may differ from previous studies of patients with cholesteatoma (7,8) because of characteristics of our patient cohort.

Several factors might have influenced our findings, including the cohort size, racial homogeneity, and the absence of diverse populations. The majority of our study population identified as White, non-Hispanic, and English-speaking, which limits the generalizability of the results to a heterogeneous population. Additionally, patients who did not undergo surgical intervention for cholesteatoma were excluded from the study because one of our primary outcome measures was to assess the delivery of and outcomes of surgery. It is possible that SDOH influences patient’s ability to pursue surgical intervention, and by excluding these patients, we have limited our ability to analyze the influence of SDOH on simply receiving recommended care. This may be a point of future study. Moreover, despite identifying 237 adult patients diagnosed with cholesteatoma, only 75 had completed preoperative SDOH questionnaires. This selection may inherently differ from those who did not complete questionnaires, making it challenging to estimate the impact of these differences. Furthermore, we did not collect data on a single chief complaint due to the heterogeneity of documentation and the challenge of identifying a single complaint to analyze amongst a constellation of symptoms. We were therefore unable to analyze any relationship between presenting symptoms and the length of time from symptom onset to seeking care. Our study did not observe significant postoperative improvements in ABG and noted relatively low rates of ossicular discontinuity and mastoid involvement by cholesteatoma, suggesting that our cohort may primarily represent early-stage cholesteatoma cases. Therefore, future research should aim to investigate the impact of SDOH on cholesteatoma treatment outcomes among diverse patient populations and across varying degrees of disease severity.

## CONCLUSIONS

Data on the relationship between SDOH and cholesteatoma care is limited. Although previous studies suggest that social factors may influence the number of surgeries, rates of postoperative residual cholesteatoma, and the invasiveness of surgery, our findings indicate a lack of association between having at least one SDOH risk factor (including stress, housing instability, financial resource strain, social connectedness, food insecurity, and intimate partner violence) and the access to, delivery of, and outcomes of cholesteatoma care. Despite stress being a common SDOH risk factor among patients with cholesteatoma, it likely does not affect access to and outcomes of care. Furthermore, while female sex, younger age, and lower education levels are associated with the presence of at least one SDOH risk factor, these demographic factors do not seem to influence cholesteatoma care outcomes. Conducting rigorous research with larger and more diverse populations is crucial to confirm these findings and to address potential disparities to improve individual health outcomes.

## ACKNOWLEDGMENTS

None declared.

## FUNDING SOURCES

None declared.

## CONFLICT OF INTEREST

The author discloses no conflicts of interest.

## DATA AVAILABILITY

Not available.

## Supplementary Material


