# Supplementary material for: The Relationship Between Social Determinants of Health and Cholesteatoma Care
Source: Otol Neurotol Open. 2025 Apr 17;5(2):e069. doi: 10.1097/ONO.0000000000000069 (PMC12208641; doi:10.1097/ONO.0000000000000069)
Supplement: Supplementary file 1 [file ono-5-e069-s001.pdf]

Supplementary Table 1. Socioeconomic Determinants of Health (SDOH) Questionnaire Data

SDOH questionnaires	
Social Connections<br><br>• Do you belong to any clubs or organizations such as church groups, unions, fraternal or athletic groups, or school groups?<br>• Do you attend meetings of the clubs or organizations you belong to?<br>• Do you get together with friends or relatives?<br>• Do you attend church or religious services?	Yes or No
Stress<br><br>• Do you feel stress-tense, restless, nervous, or anxious, or unable to sleep at night because your mind is troubled all the time-these days?<br><br>Available answers:<br>Not at all; only a little; to some extent; rather much, very much<br>Answers converted to low, medium, and high risk:<br>Not at all and only a little (low); to some extent (medium); rather much and very much (high)	Yes or No*<br><br>Low<br>Medium<br>High
Financial Resource Strain<br><br>• How hard is it for you to pay for the very basics like food, housing, medical care, and heating?<br><br>Available answers:<br>Not hard at all; not very hard; somewhat hard; hard and very hard<br>Answers converted to low, medium, and high risk:<br>Not hard at all and not very hard (low); somewhat hard (medium); hard and very hard (high)	Yes or No*<br><br>Low<br>Medium<br>High
Food Insecurity<br><br>• Within the past 12 months, you worried that your food would run out before you got money to buy more.<br>• Within the past 12 months, the food you bought just didn't last and you didn't have money to get more.<br><br>Available answers:<br>Never true; sometimes true; often true<br>Answers converted to low, medium, and high risk:<br>Never true (low); sometimes true (medium); often true (high)	Yes or No*<br><br>Low<br>Medium<br>High
Housing Stability	Yes or No*

• In the last 12 months, was there a time when you were not able to pay the mortgage or rent on time?<br>• In the last 12 months, how many places have you lived?<br>• In the last 12 months, was there a time when you did not have a steady place to sleep or slept in a shelter (including now)?<br><br>Available answers:<br>Able to pay and steady place to live and <3 places lived; sometimes not able to pay and steady place to live and <3 places lived; not able to pay or no steady place to sleep or lived in 3 or more places<br>Answers converted to low, medium, and high risk:<br>Able to pay and steady place to live and <3 places lived (low); sometimes not able to pay and steady place to live and <3 places lived (medium); not able to pay or no steady place to sleep or lived in 3 or more places (high)	Low<br>Medium<br>High
Transportation Needs	Yes or No*
• In the past 12 months, has lack of transportation kept you from medical appointments or from getting medications?<br>• In the past 12 months, has lack of transportation kept you from meetings, work, or getting things needed for daily living?<br><br>Available answer:<br>Medical transport needs met, and work/ADL transport needs met<br>Sometimes medical transport needs unmet and sometimes work/ADL transport needs unmet<br>Medical transport needs unmet and work/ADL transport needs unmet<br>Answers converted to low, medium, and high risk:<br>Medical transport needs met, and work/ADL transport needs met (low)<br>Sometimes medical transport needs unmet and sometimes work/ADL transport needs unmet (medium)<br>Medical transport needs unmet and work/ADL transport needs unmet (high)	Low<br>Medium<br>High
Intimate Partner Violence	Yes or No*
• Within the last year, have you been afraid of your partner or ex-partner?<br>• Within the last year, have you been afraid of your partner or ex-partner?<br>• Within the last year, have you been humiliated or emotionally abused in other ways by your partner or ex-partner?<br>• Within the last year, have you been kicked, hit, slapped, or otherwise physically hurt by your partner or ex-partner?<br>• Within the last year, have you been raped or forced to have any kind of sexual activity by your partner or ex-partner?<br><br>Available answers:<br>Yes or No<br>Answers converted to low, and high risk:<br>Answered all no to above questions (low)	

Answered yes to any above questions (high)	

*If patient reported yes then SDOH risk factors were classified as low risk, medium risk, or high risk.
